# Validity and Reliability Study of the Turkish Adaptation of Taste and Smell Dysfunction Questionnaire in Hemodialysis Patients

**DOI:** 10.7759/cureus.30864

**Published:** 2022-10-30

**Authors:** Yücel Kurt, Hürmüs Kuzgun

**Affiliations:** 1 Otolaryngology - Head and Neck Surgery, Finike State Hospital, Antalya, TUR; 2 Nursing, Instıtute of Health Sciences, Sakarya University, Sakarya, TUR

**Keywords:** hemodialysis, reliability, validity, smell alterations, taste alterations

## Abstract

Aim: This study was conducted to assess the validity and reliability of the Turkish adaptation of the Taste and Smell Dysfunction Questionnaire (TSDQ) in hemodialysis patients.

Materials and methods: This study involves 125 hemodialysis patients. Research data were collected using the Descriptive Characteristics Form for Hemodialysis Patients, the TSDQ, and the Questionnaire for the Assessment of Self-Reported Olfactory Functioning and Olfaction-Related Quality of Life. In addition, the questionnaire's language and content validity, construct validity, and reliability were evaluated.

Results: With regard to content validity, a high degree of agreement was determined between expert opinions on the questionnaire items (CGI = 0.96). Exploratory factor analysis revealed that the questionnaire has a two-factor structure explaining 79.33% of the total variance. The confirmatory factor analysis demonstrated that the model is within acceptable fit index limits with factor loads between 0.692 and 0.964. The Cronbach's alpha reliability coefficients of the scale and its two sub-dimensions are 0.928, 0.968, and 0.782, respectively.

Conclusion: The TSDQ is a valid and reliable tool for evaluating taste and smell changes in hemodialysis patients.

## Introduction

Although changes in smell and taste are more common among the elderly, it has been reported that they increase in several chronic diseases, affecting nutrition, safety, quality of life, and physical and mental health [1,2]. In addition, food has significant symbolic, cultural, and religious values that can affect not only nutrition but also the psychological and social aspects of life. The changes experienced by patients in taste and smell can alter their food enjoyment, dietary preferences, and food intake, resulting in chronic unintentional weight loss and malnutrition [3].

End-stage renal disease (ESRD) patients frequently exhibit characteristic oral symptoms such as taste changes, loss of taste, and dry mouth due to their treatments [4]. These symptoms often arise from disease-specific metabolic and physiological disorders. Studies have drawn attention to the relationship between dialysis treatment duration and oral lesion development [5]. In uremic patients, urea, dimethyl, trimethylamine, and low zinc levels have been associated with decreased taste perception. It has been reported that the metallic taste experienced by uremic patients is due to the saliva's urea content and its subsequent breakdown into ammonia and carbon dioxide via bacterial urease [6]. Taste disturbances (dysgeusia) in ESRD patients are generally associated with ageusia, a complete lack of taste, and hypogeusia with decreased taste sensitivity [7].

Studies suggest that olfactory identification is affected in ESRD patients. Moreover, olfactory dysfunction in patients with kidney disease is associated with the severity of the underlying disease [8,9]. Possible explanations for the occurrence of olfactory disorders include decreased capacity and impaired olfactory epithelial cell regeneration in the presence of uremic toxins [10-12].

Studies have highlighted the association between changes in the sense of taste and smell and food avoidance and anorexia in ESRD patients [13-15]. In addition, these symptoms contribute to malnutrition, one of the main determinants of morbidity and mortality in patients with kidney disease [14]. Therefore, healthcare professionals should routinely evaluate ESRD patients for changes in taste and smell. Taste and smell changes are tested using objective and subjective methods. Gustometry and electrogustometry evaluate taste disorders objectively. The sense of smell is assessed using the Sniffin' sticks test, the University of Pennsylvania Smell Identification Test, or techniques that involve inhaling solutions such as phenyl methyl-ethyl-carbinol, phenethyl, and menthol. Subjective methods include surveys, interviews, and scales [16]. In clinical practice, a subjective measurement tool that evaluates taste and smell changes together is valuable in terms of practicality and cost-effectiveness. However, when the literature was reviewed, no tool assessing taste and smell changes together was encountered in Turkey. Therefore, this study was conducted to ensure the validity and reliability of the Turkish adaptation of the Taste and Smell Dysfunction Questionnaire (TSDQ) in hemodialysis patients to address the abovementioned gap in the literature.

## Materials and methods

Study design and sample

This study methodologically evaluates the validity and reliability of the Taste and Smell Dysfunction Questionnaire in hemodialysis patients. Data were collected in the hemodialysis centers of two public hospitals in the districts of Antalya between July and August 2022. The study population comprised patients receiving hemodialysis (N = 135). The study sample included 125 patients who volunteered to participate in the research and met the inclusion criteria. The study participants were selected using non-probability sampling. When determining the sample size, care was taken to ensure that it was at least five times the number of questionnaire items, as recommended for methodological studies [17,18]. The inclusion criteria were being 18 years or above, receiving hemodialysis treatment for at least six months, not having a psychiatric diagnosis, and volunteering to participate in the study.

Data collection

The research data were collected from patients during dialysis treatment using face-to-face interviews. The patients filled out the forms within 15-25 minutes.

The data were collected using the Descriptive Characteristics Form for Hemodialysis Patients, the TSDQ, and the 12-item Questionnaire for the Assessment of Self-Reported Olfactory Functioning and Olfaction-Related Quality of Life.

The Descriptive Characteristics Form for Hemodialysis Patients includes questions evaluating descriptive characteristics of patients such as age, gender, marital status, educational status, occupation, year of starting hemodialysis, primary kidney disease, and accompanying chronic diseases.

The TSDQ was developed by Heald et al. in 1998 to evaluate changes in taste and smell in HIV patients. Later on, this questionnaire was used in different disease groups (patients with various types of cancer, irritable bowel syndrome, neurological diseases, and the elderly) [19-22]. The TSDQ is a 16-item scale used to determine an individual's perceived and reported changes in taste and smell. It includes 11 questions about taste changes and five about smell changes. The questions related to the sense of taste concern whether there is a perceived change (strong or weak) in salty, sweet, sour, and bitter tastes and the extent of this change. Likewise, the questions related to the sense of smell concern whether there is a perceived change in the smell of a particular food, whether it smells stronger or weaker, and the extent of this change (insignificant, mild, moderate, or unbearable) [22]. Items 6, 8, 10, 14, and 16 are not included in scoring in the original version of the questionnaire, and item 11 is calculated as four sub-items: 11a, 11b, 11c, and 11d. The total score that can be received from nine questions that address perceived changes in the sense of taste ranges from 0 to 10. The total score that can be obtained from five questions on perceived smell changes ranges from 0 to 6. The sum total of the taste and smell change scores ranges from 0 to 16. The higher the score, the greater the complaints about taste and smell changes.

The 12-item Questionnaire for the Assessment of Self-Reported Olfactory Functioning and Olfaction-Related Quality of Life was developed by Pusswald et al. (2012) and adapted into Turkish by Saatci et al. (2020) [23,24]. This survey comprises three areas: the overall olfactory capacity reported by the patient (consisting of one item), the patient's reported capacity to detect certain odors (consisting of five items), and the reported olfaction-related quality of life of the patient (consisting of six items). A score of ≤3 received from the first area indicates abnormal olfactory ability. A score of ≤2.9 from the second area indicates problems with the sense of smell. A score of ≤3.7 from the third area indicates that the smell adversely affects the patient's quality of life [24]. In the scale's adaptation study, Cronbach's alpha reliability coefficient for the sub-dimensions was 0.97-0.98. In this study, it was determined as 0.916-0.971.

Statistical analysis

The study data were evaluated using SPSS 23.0 (SPSS Inc, Chicago, Illinois) and AMOS 24.0. The descriptive statistics used to analyze the socio-demographic data were percentage, frequency, median, minimum-maximum values, mean, and standard deviation. In order to determine the reliability of TSDQ, Cronbach's alpha values were calculated by determining the item-total score correlation coefficients of 14 items and conducting the internal consistency analysis of the items in the scale. In order to assess the scale's invariance over time, a test-retest (retest) application was carried out with 20 participants who were contacted three weeks after the first application. The Pearson product-moment correlation coefficient was calculated for the test-retest. The questionnaire's validity was evaluated using the concurrent validity approach from content validity, construct validity, and criterion-related validity approaches. Content validity requires subjecting the scale to expert opinion. The content validity index was calculated to determine the scale's content validity. Exploratory factor analysis (EFA) and confirmatory factor analysis (CFA) were applied to evaluate the scale's construct validity. Principal component analysis was used in the EFA. Bartlett's Test of Sphericity and Keiser-Mayer-Olkin Test were applied to determine the adequacy of scale content and sample size. The questionnaire's factor structure and factor loads were examined using CFA. A path diagram of the questionnaire was created using AMOS software. For criterion-related validity, Pearson correlation analysis was performed between the TSDQ and the 12-item Questionnaire for the Assessment of Self-Reported Olfactory Functioning and Olfaction-Related Quality of Life.

Ethical considerations

Before commencing the study, approval was obtained from a university's non-interventional Clinical Research Ethics Committee (Protocol no: 2022/85) and from the institution where the study was conducted. Furthermore, the study's purpose was explained to the hemodialysis patients verbally and in writing before obtaining their consent. Finally, permission was obtained from the author who developed the TSDQ to adapt it into Turkish and for the other survey to be used as a parallel form.

## Results

The mean age of the 125 hemodialysis patients participating in the study was 61.03 ± 10.99 (min = 20, max = 96). Of the study participants, 56.8% were female, 60.8% were secondary school graduates, 76.0% were married, 36.0% had an initial diagnosis of hypertensive kidney disease, and 67.2% had no comorbidities. The patients had received dialysis treatment for an average of 5.25 ± 4.13 years (Table 1).

**Table 1:** Characteristics

Characteristics		n = 125	%
Gender	Male	54	43.2
	Female	71	56.8
Education	Literate	15	12.0
	Primary school	7	5.6
	Secondary school	76	60.8
	High school	18	14.4
	University	9	7.2
Marital Status	Married	95	76.0
	Single	30	24.0
Initial Diagnosis	Chronic glomerulonephritis	35	16.8
	Hypertensive kidney disease	45	36.0
	Pyelonephritis	6	4.8
	Diabetic nephropathy	42	33.6
	Polycystic kidney disease	11	8.8
Existence of Additional Diseases	Yes	41	32.8
	No	84	67.2
	X ± SD (Min-Max)		
Age (years)	61.03 ± 10.99 (20–96)		
Years of Hemodialysis	5.25 ± 4.13 (0.5–18)		

Language validity

Two independent translators proficient in both languages translated the scale from English into Turkish in the language validity step. Two individuals fluent in English combined and arranged the two scales into a single tool. The translation, which is the final version in Turkish, was back-translated from Turkish into English by an expert proficient in both languages. The questionnaire back-translated into English was compared with the original version in English. No change was observed in the meaning of the scale items in this comparison; thus, the scale's language validity was confirmed.

Content validity

Seven experts evaluated the questionnaire's original English version and Turkish translation to ensure content validity using the Davis technique [25]. As a result, the draft scale's content validity index values were found to be 0.96 on average and to range from 0.70 to 1.00.

Pilot application

Expert suggestions were evaluated, and changes were made accordingly. The scale was then presented to a group of 10 individuals. Since the participants found each item understandable, no changes were made.

Construct validity

The sample's sufficiency was assessed using the Kaiser-Meyer-Olkin (KMO) sampling adequacy test. Bartlett's Test of Sphericity was used to determine the suitability of the factor correlation matrix. The KMO result was determined as 0.907, and Bartlett's Test of Sphericity result as x2 = 1,869.745 (p = 0.000). The EFA to determine the number of TSDQ subscales revealed that TSDQ had a two-factor structure with an Eigen-value of above 1.00. The relationship structure between the factors was ensured to remain the same using the Varimax method in factor analysis, and it was determined that it explained 79.337% of the variance. The TSDQ items were kept the same as in the original scale (Table 2).

**Table 2:** Distribution of TSDQ item scores and item-total correlations TSDQ = Taste and Smell Dysfunction Questionnaire, no reverse scoring; SD = standard deviation; r = item-total correlation

Scale sub-dimensions		X ± SD	Factor load	Explained variance	r	Cronbach's alpha
Taste	T3	0.376 ± 0.486	0.945	51.030	0.902	0.968
	T11b	0.424 ± 0.496	0.926		0.934	
	T1	0.432 ± 0.497	0.921		0.952	
	T11d	0.424 ± 0.496	0.878		0.876	
	T7	0.424 ± 0.496	0.877		0.939	
	T11c	0.448 ± 0.499	0.853		0.835	
	T11a	0.440 ± 0.498	0.809		0.817	
	T13	0.688 ± 0.776	-0.808		0.848	
	T5	2.32 ± 1.388	-0.769		0.817	
Taste Total	5.60 ± 4.85					
Smell	T12	0.5680 ± 0.497	0.976	28.307	0.704	0.782
	T10	0.632 ± 0.757	0.971		0.940	
	T2	1.456 ± 0.701	0.896		0.678	
	T9	0.536 ± 0.838	0.888		0.956	
	T4	0.656 ± 0.476	0.481		0.710	
Smell Total	3.60 ± 1.507					
TSDQ	9.28 ± 5.67			79.337		0.928

**Figure 1:**
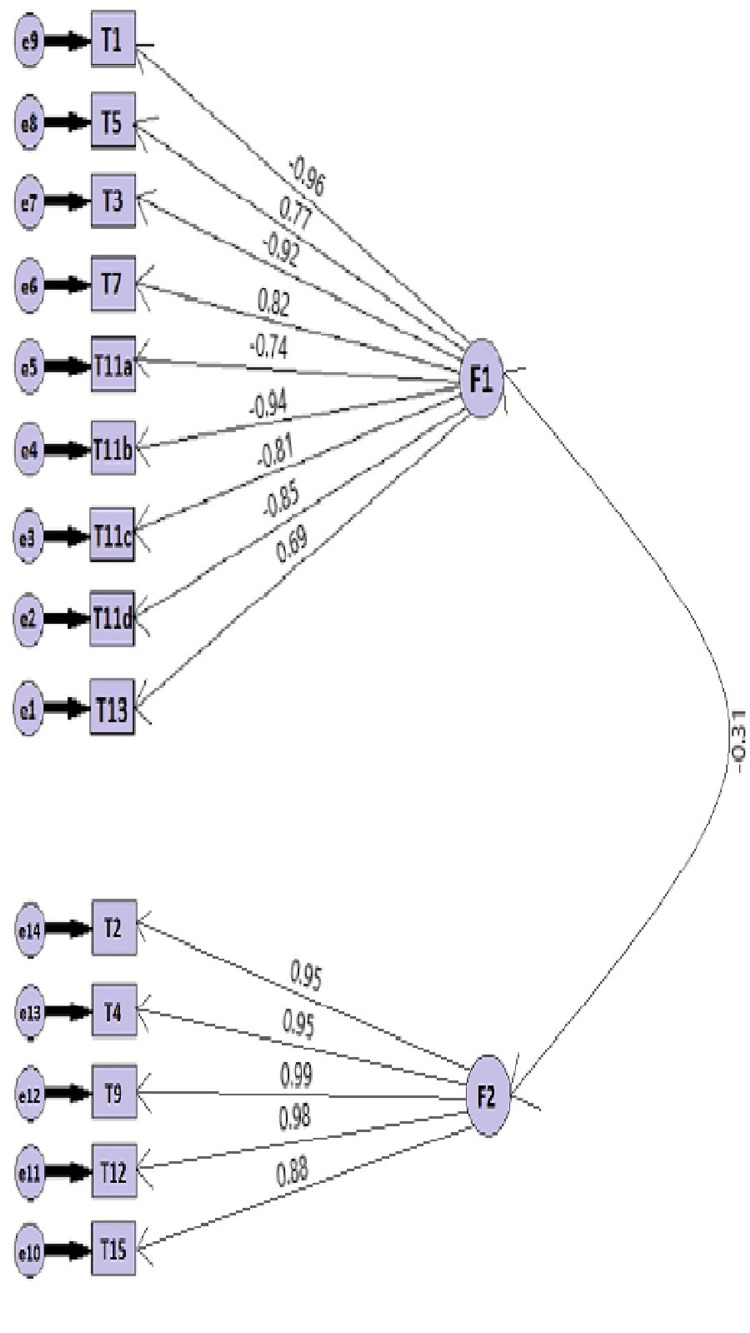
Path diagram

Confirmatory factor analysis was conducted to evaluate the content validity in the Turkish adaptation of the TSDQ. As a result, the model was seen to fall within the limits of the Goodness of Fit Index (GFI) as the RMSEA value of the model was 0.912, the chi-square value was statistically significant (χ2 = 219.912; n = 68, SD = 76, p = 0.00, χ2/SD = 2.894), the Comparative Fit Index (CFI) was 0.912, and the GFI was 0.902. The path diagram produced through confirmatory factor analysis is presented in Figure 1. The factor loads of the scale items range from 0.69 to 0.96.

When the correlation between two similar scales was reviewed for criterion-related validity, the Pearson correlation coefficient between the TSDQ and Questionnaire for the Assessment of Self-Reported Olfactory Functioning and Olfaction-Related Quality of Life was calculated as 0.430 (Table 3).

**Table 3:** Correlation between TSDQ and Questionnaire for the Assessment of Self-Reported Olfactory Functioning and Olfaction-Related Quality of Life TSDQ = Taste and Smell Dysfunction Questionnaire

	Taste	Smell	TSDQ
Smell	r = 0.322	r = 0.512	r = 0.422
	p = 0.000	p = 0.000	p = 0.000
Taste	r = 0.331	r = 0.523	r = 0.461
	p = 0.000	p = 0.000	p = 0.000
Questionnaire for the Assessment of Self-Reported Olfactory Functioning and Olfaction-Related Quality of Life	r = 0.312	r = 0.541	r = 0.430
	p = 0.000	p = 0.000	p = 0.000

Reliability

The Pearson product-moment correlation analysis was applied to evaluate the compatibility between the test-retest mean scores of the TSDQ. A statistically significant strong positive correlation was observed between the two measurements in the overall scale and the sub-dimensions (Table 4). The difference between the scores obtained from the draft questionnaire's two measurements performed at a three-week interval was evaluated through a t-test in dependent groups. No statistically significant differences were observed between the two measurements (Table 4).

**Table 4:** TSDQ test-retest analysis T-test= Paired sample t-test; r= Correlation between two measurements; pa=  p-value of the t-test; pb=  p-value of the correlation. TSDQ = Taste and Smell Dysfunction Questionnaire

	Substances	N	Test	Retest	t-test	p^a^	r	p^b^
			X ± SD	X ± SD				
Taste	9	15	5.97 ± 4.99	5.63 ± 4.52	0.582	0.502	0.925	0.00
Smell	5		3.21 ± 1.49	3.15 ± 1.52	0.624	0.472	0.919	0.00
TSDQ Total	14		9.19 ± 5.69	9.20 ± 5.76	0.602	0.489	0.921	0.00

As items 6,8,10,14, and 16 were not included in the scoring of the original version of the questionnaire, they were also excluded from the analysis in this study. In addition, item 11 in the questionnaire was calculated as four sub-items 11a, 11b, 11c, and 11d. Thus, 14 items of TSDQ were included in the analysis as in the original version of the questionnaire. According to the correlation-based item analysis result, the item sub-dimension total score correlation coefficients for the 14 items range from 0.817 to 0.952 in the taste sub-dimension and 0.678 to 0.956 in the smell sub-dimension (Table 2).

Cronbach's alpha internal consistency coefficient was calculated to evaluate the TSDQ's reliability. The scale's overall reliability is α=0.968 in the taste sub-dimension, α=0.782 in the smell sub-dimension, and α=0.928 in the total TSDQ (Table 2). The mean scores obtained from the scale were calculated to be 5.60 ± 4.85 in the taste sub-dimension, 3.60 ± 1.507 in the smell sub-dimension, and 9.28 ± 5.67 in total.

## Discussion

This study was conducted to assess the validity and reliability of the Turkish adaptation of 16-item TSDQ, which was developed to evaluate changes in taste and smell. In this context, the language, content, construct validity, and reliability of TSDQ were analyzed. In line with the analyses conducted, the scale's original form was preserved, and the scale items were kept unchanged.

According to the wider literature, the content validity index should be at least 0.80 [25]. In the content analysis conducted in this study, this index was determined to be relatively high (0.96). Hence, the scale meets the criteria for content validity.

The KMO coefficient and Bartlett's Test of Sphericity are generally used to test the scale's adequacy in terms of construct validity [17,18,26]. The wider literature reports that when the factor analysis method is to be used, the KMO value needs be at least 0.50 as a result of the sample adequacy test. A KMO value between 0.70 and 0.80 indicates moderate sample adequacy, a value between 0.80 and 0.90 indicates acceptable sample adequacy, and a value greater than 0.90 indicates excellent sample adequacy [27]. The fact that Bartlett's Test of Sphericity result is significant indicates that the scale items' correlation matrix is suitable for factor analysis [17,26]. In this study, the KMO coefficient and Barlett's Test of Sphericity were applied to determine the scale's adequacy in terms of construct validity. The KMO value was determined to be 0.907, and the Barlett test result was found to be significant (p = 0.000). Thus, it was determined that the research sample size was sufficient for factor analysis, and factor analysis could be applied to the scale.

When evaluating the EFA results, it is necessary to consider whether the scale has a single- or multi-factor structure. According to the wider literature, at least 30% of the total variance should be able to be explained in single-factor scales, and this figure is expected to be higher for multi-factor scales [28]. The EFA results in this study indicate that the scale has a two-factor structure that explains 79.33% of the variance (Table 2). The scale consists of two areas in the original version and the Swedish adaptation [21].

The questionnaire's factor loads and fit index values were assessed using CFA. The wider literature recommends a factor load value of at least 0.30 for scale items; items below this value should be removed from the scale [18,29,30]. TSDQ factor loads were found to be between 0.69 and 0.96 in the CFA (Figure 1). Therefore, no items were removed from the scale.

The CFA applied in this study supports the two-factor structure obtained through the EFA. An examination of the model's goodness of fit revealed an RMSEA of 0.91, a statistically significant chi-square value (χ2 = 219.912; n = 68, SD = 76, p = 0.00, χ2/SD = 2.894), a CFI of 0.912, and a GFI of 0.902. In the wider literature, an χ2/SD ratio of ≤5, RMSEA of ≤0.08, and GFI, CFI, and IFI (Incremental Fit Index) values above 0.90 indicate that the model is within acceptable limits [17,18,29,30].

This approach compares the scale with a similar scale priorly assessed for validity and reliability [17]. This study examined the relationship between the TSDQ and the Questionnaire for the Assessment of Self-Reported Olfactory Functioning and Olfaction-Related Quality of Life. As expected, a moderate positive correlation was observed between the two. This finding supports the assumption that the TSDQ is a valid scale.

One of the reliability tests is based on assessing the scale's test-retest results [17,26]. This method compares the correlation coefficient between the measurements made twice at a certain interval in the same group to evaluate the test measurements' stability over time. A strong correlation demonstrates that the test scores are stable and the measured property has not changed excessively between the two measurements depending on time [17]. In this study, the correlation coefficient (0.921) between the TSDQ test-retest scores was determined to be very high (Table 4). According to these findings, the scale can be considered a consistent measurement tool in the face of time.

In order to determine the adapted questionnaire's reliability, Cronbach's alpha values were determined by calculating the item-total score correlation coefficients and analyzing the internal consistency of the items included in the questionnaire (Table 2). The item-total core correlation was seen to range between 0.817 and 0.952 in the taste sub-dimension and between 0.678 and 0.956 in the smell sub-dimension. Although opinions about the item-total score correlation coefficient differ in the wider literature, 0.20 is generally accepted as the lower limit. Items with a correlation coefficient between 0.30 and 0.40 are assumed to be good, and items with a correlation coefficient above 0.40 are considered very good [18,26].

In this study, Cronbach's alpha values for the TSDQ sub-dimensions were 0.968 and 0.782. The TSDQ's overall reliability was α=0.928 (Table 2). In the Swedish adaptation of the scale, Cronbach's alpha values for the sub-dimensions were 0.79-0.64, and its overall reliability was α=0.71 [21]. In the wider literature, Cronbach's alpha coefficient is reported to vary between 0.0 and 1.0. The scale is reasonably reliable if the coefficient is between 0.60 and 0.80 and highly reliable if the coefficient is 0.80 or above [17]. The Cronbach's alpha value calculated in our study indicates that the scale is highly reliable in line with the wider literature.

This study has several strengths and weaknesses. In the present study, the validity and reliability of the Turkish adaptation of TSDQ were investigated only in hemodialysis patients. This is a considerable limitation. Therefore it is recommended that the scale be studied in different chronic patient groups whose taste and smell could be affected. In addition, our study was conducted using data obtained from two local hospitals. Therefore, we recommend conducting the study in multiple centers with larger populations.

## Conclusions

This study is the first to evaluate the TSDQ's psychometric properties in Turkey. The findings are consistent with the results obtained from analyses of the original scale. The EFA and CFA results demonstrated that the scale has two factors. In addition, Cronbach's alpha internal consistency coefficient, item-total correlation, and test-retest analysis revealed the high reliability of TSDQ. These results indicate that the TSDQ is a valid and reliable instrument for evaluating hemodialysis patients' taste and smell changes.
